# The Colonization of Epileptics

**Published:** 1897-02

**Authors:** 


### THE COLONIZATION OF EPILEPTICS.

Two years ago New York State established a colony for epileptics modeled upon the similar colony at Bielefeld, Germany. The manifest object of this wise and humane legislation was to get these unfortunate people out of the asylums and almshouses, and to give them a home; to provide them with schools and industrial training, and to give each case careful scientific study and treatment.

The institution was opened for the reception of patients February 1st, 1896, at Sonyea, New York, upon a reservation of 1,872 acres of land, part of which lay in the fertile and famous Genesee Valley. The first report of the colony has just been made, and we have also before us an interesting paper by the medical superintendent, Dr. Spratling, upon "What the Colony System Means in the Care and Treatment of Epileptics."*

These papers show eloquently what judicious and scientific care and management may accomplish for epileptic patients in a colony. In the colony the epileptic is given something to do; he is given an occupation; he is made productive. Those whose physical condition admit of it are put at light manual labor, and not only does this supply one of the important features in the proper management of the epileptic, but each, from an economic point of view, is made to a great degree, self-supporting. In the first eight months embraced by the report 145 patients were admitted. "The first fifty cases received had during their first month at the colony 708 seizures; the same fifty cases, after five months of treatment, had collectively during the fifth month 315 seizures." "Nearly every individual markedly improved physically and gained weight. Their epileptic seizures have diminished in frequency to a noteworthy degree, and in a few

*Albany (N. Y.) *Medical Annals*, January, 1897.

instances there has been a complete cessation of attacks for months at a time. . . . A strong evidence of the appreciation of the colony by the patients is afforded by the fact that although held there by no legal form of commitment, but having perfect liberty of the premises, they rarely manifest the least desire to leave. . . . The colony has produced about 50 per cent. of the cost of maintenance. It is expected that this percentage will be increased as the industrial facilities are enlarged."

We are sure that New York's experience with her Craig Colony for epileptics should encourage other States to adopt this initiative in the proper and humane care of their epileptics. A well conducted colony in every State would give these people a home instead of an asylum; it would give them employment in the place of alms.

